# Long-term safety and effectiveness of romiplostim for chronic idiopathic thrombocytopenic purpura in real-world settings

**DOI:** 10.1007/s12185-024-03847-4

**Published:** 2024-09-29

**Authors:** Naoshi Obara, Shigeki Hatanaka, Yukie Tsuji, Koji Higashi

**Affiliations:** 1 Department of Hematology, Faculty of Medicine, University of Tsukuba, Tennodai 1-1-1, Tsukuba, Ibaraki 305-8575 Japan; 2 Pharmacovigilance Division, Kyowa Kirin Co., Ltd., Tokyo, Japan

**Keywords:** Chronic idiopathic thrombocytopenic purpura, Romiplostim, Specified Use-Results Survey, Thrombopoietin, Japan

## Abstract

Idiopathic thrombocytopenic purpura (ITP), an autoimmune hematologic disorder characterized by severe platelet count reduction, can be treated with romiplostim. However, post-marketing safety and effectiveness data for romiplostim in Japan are scarce. This prospective, observational, post-marketing Specified Use-Results Survey evaluated the real-world safety and effectiveness of romiplostim for 2 years. All patients treated with romiplostim during the survey period were eligible. Of the 1622 patients in the safety analysis set, 94.08% (1526/1622) had chronic ITP. The mean single dose of romiplostim was stable after 12 weeks and remained < 6 μg/kg in approximately 70% of patients until 104 weeks. Within 2 years, 14.92% of patients discontinued romiplostim because of adverse events, while 6.47% discontinued because of suspected adverse drug reactions. In contrast, 14.00% of patients discontinued romiplostim because of symptom improvement. Before romiplostim initiation, platelet count was < 2.0 × 10^4^/µL in 60.54% of patients, and the mean platelet count was 2.84 ± 5.76 × 10^4^/µL. Platelet count was 9.19 ± 13.01 × 10^4^/µL after 4 weeks, and remained between 10.34 ± 10.72 and 12.38 ± 12.63 × 10^4^/µL from 8 to 104 weeks of treatment. No specific concerns were revealed regarding the safety and effectiveness of romiplostim in chronic ITP; the findings demonstrated a favorable risk–benefit balance for romiplostim in this population. *Trial registration*: UMIN000047864 (www.umin.ac.jp/ctr).

**Supplementary Information:**

The online version contains supplementary material available at 10.1007/s12185-024-03847-4.

## Introduction

Idiopathic thrombocytopenic purpura (ITP) is a commonly occurring autoimmune haematologic disorder characterised by a severe reduction in platelet count, for which the ideal treatment is to increase platelet count to a safe haemostatic range [1, 2]. Romiplostim (Romiplate^®^, Kyowa Kirin Co., Ltd., Tokyo, Japan) is a thrombopoietin receptor agonist (TPO-RA) that binds to TPO receptors on megakaryocytes and their progenitor cells in bone marrow, thereby enhancing platelet production. It was first approved in 2008 in Australia, and in the same year in the USA, for treating chronic ITP in patients with prior unfavourable responses to corticosteroids, immunoglobulins, or splenectomy and in those with a higher risk of haemorrhage due to the degree of thrombocytopenia [3]; it was subsequently approved in Japan in 2011 [4]. Regulatory authorities in Japan requested that additional information on the effectiveness and safety of all patients treated with the product be acquired through post-marketing surveillance as conditions for approval.

Multiple studies have already established the efficacy and safety of romiplostim in patients with chronic ITP [5, 6]. In Japanese adult patients with chronic ITP, a significantly higher platelet response was found after 12 weeks of treatment with romiplostim when compared with placebo (*P* < 0.0001) [5]. Moreover, a significantly higher proportion of patients achieved a sustained platelet response after 24 weeks of romiplostim treatment than those receiving a placebo among those with or without a history of splenectomy [6]. The efficacy and safety of long-term romiplostim administration in patients with chronic ITP have also been reported [7, 8]. The safety of romiplostim was evaluated in 13 domestic and international clinical trials, which were the basis for the approval in Japan [9, 10]. The Japanese long-term extension study of romiplostim was conducted in only a few patients (*n* = 44) [8], offering limited clinical data. In addition, based on the results of a phase 2 clinical trial in Japan [11], romiplostim was recommended at a starting dose of 3 μg/kg for phase 3 evaluation in Japanese patients with chronic ITP, despite the approved dosage being lower (1 μg/kg) [4]. The previous studies have associated romiplostim treatment with a higher risk of increased bone marrow reticulin or myelofibrosis, haematological malignancies, and myelodysplastic syndromes (MDS) [12–14]. Therefore, there is a need to collect data pertaining to the safety of long-term romiplostim treatment (> 1 year).

There is a lack of real-world data on the effectiveness and safety of romiplostim in patients with chronic ITP, particularly in Japan. The present Romiplate^®^ for subcutaneous injection 250 µg Specified Use-Results Survey—All Patients Surveillance—“Survey on Long-term Use” was planned and started in April 2011. The objective of this survey was to evaluate the safety and effectiveness of romiplostim in clinical practice, with three specific requirements: understanding the occurrence of suspected adverse drug reactions (ADRs), detecting suspected unexpected ADRs, and assessing priority survey items (worsening of thrombocytopenia and haemorrhage-related events after discontinuation of romiplostim, haemorrhagic suspected ADRs, and thromboembolic suspected ADRs) and adverse events (AEs) of special interest (increased bone marrow reticulin or myelofibrosis, haematological malignancies, and MDS), and confirming the safety and effectiveness of romiplostim during long-term use (2 years).

## Methods

### Clinical setting and intervention

This prospective, observational, post-marketing all-patients surveillance was launched on April 13, 2011, at sites across Japan. Using a central registration system, the survey was conducted as an all-patients surveillance that examined all patients who received romiplostim during the first 5 years of romiplostim marketing. The registration period lasted 5 years, between April 2011 and the end of March 2016; in patients where romiplostim was started on or after April 1, 2016, only the registration of patients treated with romiplostim was continued. Each patient was observed for 2 years. However, those who discontinued romiplostim within the 2-year observation period were observed up to 1 month after the discontinuation.

Administration of romiplostim was based on the Japanese approval dosage [4]. The initial adult dose of 1 μg/kg of romiplostim was administered subcutaneously and then once weekly at that dosage thereafter, depending on the patient’s platelet count and other symptoms. The maximum dose allowed was 10 μg/kg once weekly. All enrolled patients had their survey forms completed by the survey site investigator at 6, 12, 18, and 24 months after starting romiplostim administration and submitted to the medical representatives. Re-survey was conducted for incomplete items. All patients treated with romiplostim were annually confirmed for registration at all survey sites using the “Confirmation of All Cases Form”.

The protocol of this survey was reviewed by the Japanese Pharmaceuticals and Medical Devices Agency (PMDA) [15]. The survey was conducted in accordance with the Good Post-Marketing Study Practice (GPSP) ordinance. Hence, the provision for written informed consent and ethics committee approval were waived for this survey. Written consent was obtained from each survey site for this publication; all but 44 sites (162 patients), consented to the publication of the results. The survey was registered with the University hospital Medical Information Network under the identifier UMIN000047864.

### Patients

All patients treated with romiplostim by subcutaneous injection for chronic ITP or diseases other than chronic ITP (off-label use) during the survey period were included according to the pre-specified protocol (Supplementary Table 1). There were no specific exclusion criteria set for this survey.

### Safety

The safety outcomes assessed in this survey included the information on the occurrence of suspected ADRs, detection of suspected unexpected ADRs, assessment of priority survey items (worsening of thrombocytopenia and haemorrhage-related events after discontinuation of romiplostim, haemorrhagic suspected ADRs, thromboembolic suspected ADRs) and AEs of special interest (increased bone marrow reticulin or myelofibrosis, haematological malignancies, and MDS).

In this survey, AEs were defined according to the International Conference on Harmonisation of Technical Requirements for Registration of Pharmaceuticals for Human Use (E2D) guideline [16] as “any unwanted or unintended signs (including abnormal laboratory values), symptoms, or illnesses occurring on the administration of romiplostim, regardless of whether or not they were causally related to romiplostim”. A suspected ADR was defined as “an AE for which a causal relationship with romiplostim could not be ruled out, including those of unknown causation”. The AEs were categorised according to the Medical Dictionary for Regulatory Activities (MedDRA), Japanese version 23.0. The survey site investigator classified the seriousness of AEs according to the Ordinance for Enforcement of the PMDA. The AEs were defined as non-serious or serious (including fatal, disability, life-threatening, potentially leading to disability, hospitalisation for treatment or prolongation of hospitalisation, other serious AEs equivalent to the previous 5 points, or congenital disease or abnormality in later generations). In cases where the sponsor determined that expedited reporting to the regulatory authorities was necessary, non-serious AEs were reclassified as serious AEs.

### Effectiveness

The effectiveness endpoint of this survey was changes in platelet count from the start of romiplostim administration to 104 weeks (2 years). Platelet counts that would affect the evaluation of romiplostim effectiveness, and therefore, not used for analysis, included those recorded within 8 weeks after platelet transfusion; during the period of new administration of steroids (excluding methylprednisolone injection ≥ 1 g) after romiplostim administration; during the period of new administration of “other drugs used for chronic ITP (eltrombopag olamine, azathioprine, cyclosporine, cyclophosphamide, danazol, mycophenolate mofetil)” after initiation of romiplostim; during the period of new *Helicobacter pylori* eradication therapy or new rituximab administration after initiation of romiplostim; after splenectomy after initiation of therapy with romiplostim; if a new dose of intravenous human immune gamma globulin was administered after the start of treatment with romiplostim, platelet count within 3 weeks after the start and end of treatment with romiplostim; if ≥ 1 g of methylprednisolone (injection) was used after the initiation of romiplostim administration, platelet count within 4 weeks from the start until the end of romiplostim therapy; or if a new dose of diphenyl sulfone or vinca alkaloids was administered after the initiation of romiplostim, platelet count within 1 week from the start until the end of treatment with romiplostim. The analysis did not include periods during which other drugs might affect platelet counts (i.e. while on medication).

### Statistical methods

The sample size for this survey was set at 700 patients. In order to detect at least one case of suspected ADR with an incidence rate of 0.5% or higher with a probability of 95% or higher in the post-marketing period, approximately 600 patients were required [17]. The target number of 700 patients was set, based on the estimated number of patients using the other drug with the same indication, which was, in turn, calculated considering the estimated number of patients using romiplostim over the 5-year period.

The safety analysis set was defined as all eligible patients receiving romiplostim, registered by the end of March 2016, for whom locked data were available. Patients who did not receive romiplostim, or those who had not received romiplostim, those with duplicate registration after the survey was collected, and those with duplicate registration who could not be evaluated for safety were excluded from the total number of registered patients.

The effectiveness analysis set included patients who were excluded from the safety analysis set: those without data on platelet counts after romiplostim treatment, those who had received romiplostim previously, or those who had received romiplostim as off-label use, i.e., for conditions other than chronic ITP.

Descriptive statistics were used to compare patient characteristics. For the safety analysis, the incidences of AEs and suspected ADRs were calculated. For the effectiveness analysis, descriptive statistics were used for outcome measurements. All statistical analyses were conducted by the contract research organization INTAGE Healthcare Inc. (now; Ark Medical Solutions Inc; Tokyo, Japan), using SAS Software version 9.4 (SAS Institute Inc., Cary, NC, USA).

## Results

### Patients

**Fig. 1:**
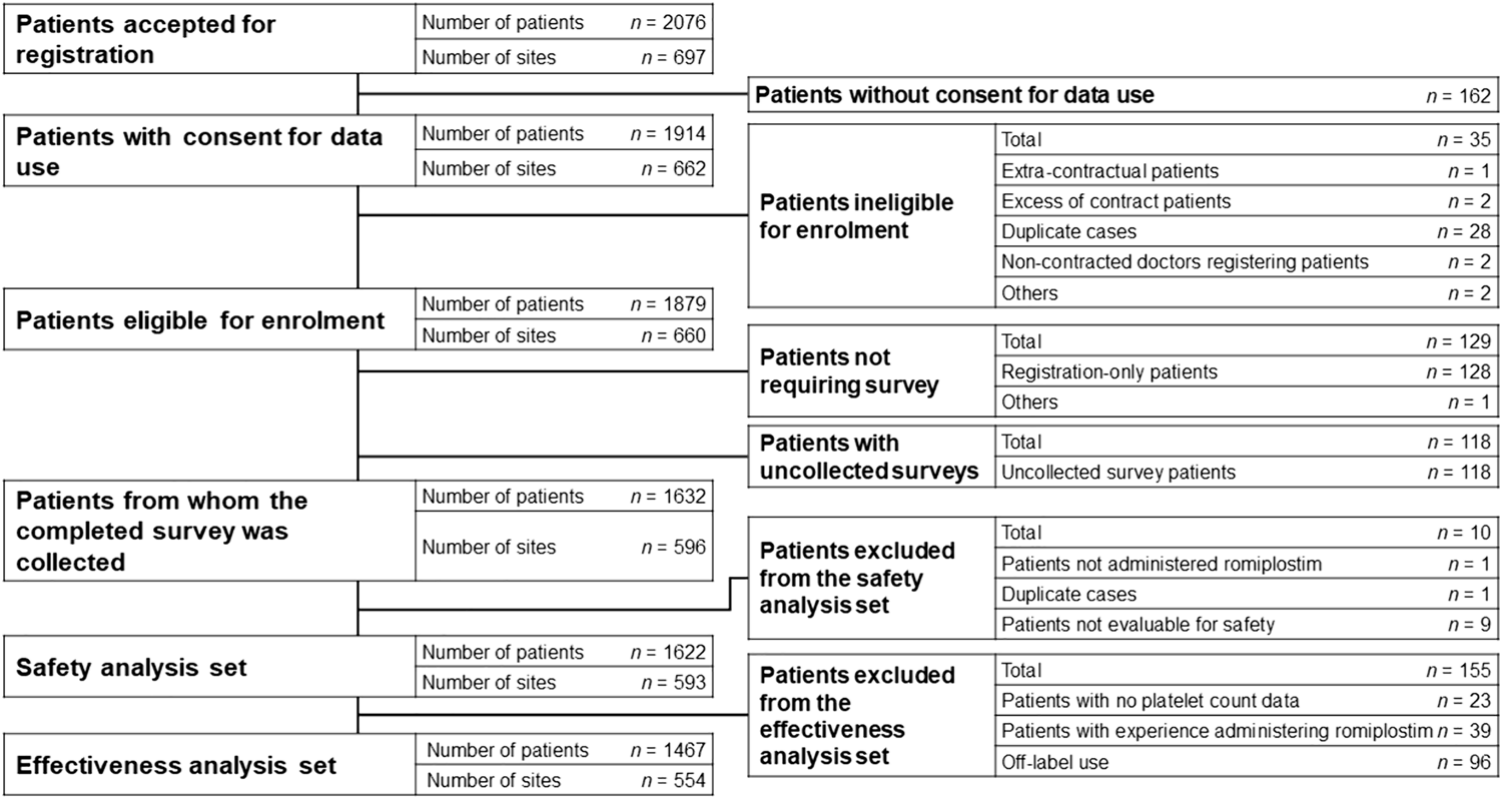
Patient disposition

The disposition of patients is displayed in Fig. 1; 2076 patients were accepted for registration at 697 sites, of which 162 did not consent to the publication of the survey results. Of the 1879 patients eligible for enrolment from 660 sites, 1632 patients completed the survey. The safety analysis set included 1622 patients, and the effectiveness analysis set included 1467 patients.

Table 1 describes the characteristics of patients in the safety analysis set at the start of treatment. At the start of romiplostim administration, 56.97% of patients were aged ≥ 65 years. Romiplostim was administered to 94.08% of patients as a treatment for chronic ITP; platelet counts were < 2.0 × 10^4^/µL in 60.54% of patients and ≥ 2.0 to < 3.0 × 10^4^/µL in 11.22%. At the time of romiplostim initiation, corticosteroids were administered in 84.40% of chronic ITP patients, and splenectomy was performed in 17.76%.

**Table 1:** Patient characteristics at the start of treatment (safety analysis set)

Safety analysis set (*n* = 1622) *n* (%)	
Sex	
Male	708 (43.65)
Female	914 (56.35)
Age, years	
< 15	51 (3.14)
≥ 15, < 65	638 (39.33)
≥ 65	924 (56.97)
Unknown	9 (0.55)
Treatment reason	
Chronic ITP	1526 (94.08)
Off-label use**	96 (5.92)
Body weight, kg	
< 54.0	785 (48.40)
≥ 54.0	801 (49.38)
Unknown	36 (2.22)
Date of onset of disease, years	
≤ 3	738 (48.36*)
> 3	577 (37.81*)
Unknown	211 (13.83*)
Bleeding symptoms	
No	350 (22.94*)
Yes	1134 (74.31*)
Unknown	42 (2.75*)
Platelet count before initiation of romiplostim, × 10^4^/µL	
< 2.0	982 (60.54)
≥ 2.0, < 3.0	182 (11.22)
≥ 3.0	403 (24.85)
Unknown	55 (3.39)
Treatment history for chronic ITP	1393 (91.28*)
*Helicobacter pylori* eradication therapy	459 (30.08*)
Corticosteroid preparations	1288 (84.40*)
Splenectomy	271 (17.76*)
Human immune gamma globulin for intravenous infusion	563 (36.89*)
Eltrombopag olamine	493 (32.31*)
Romiplostim	39 (2.56*)
Third line (based on reference guide for adult ITP [18])	214 (14.02*)
Other	11 (0.72*)

*ITP* Immune thrombocytopenia

*Chronic ITP; *n* = 1526

**ITP, thrombocytopenia, myelodysplastic syndrome, aplastic anaemia, platelet count decreased, systemic lupus erythematosus, Wiskott-Aldrich syndrome, Evans syndrome, acute myeloid leukaemia, hepatitis C, Castleman's disease, lymphoma, hepatic cirrhosis, plasma cell myeloma, platelet function test abnormal, bone marrow failure, mucinous adenocarcinoma of the appendix, disseminated intravascular coagulation, pancytopenia, and pancreatic cancer

The exposure to romiplostim in the safety analysis set is shown in Table 2. The mean single dose of romiplostim was stable after 12 weeks from the start of romiplostim administration and remained below 4 µg/kg in nearly 50% of patients; the dose remained below 6 μg/kg in approximately 70% of patients (data not shown). The median romiplostim treatment duration was 190 (range, 1–1203) days. By the end of the survey period, 226 patients had received at least one maximum dose of 10 μg/kg, and 25 patients had received at least one maximum dose of > 10 μg/kg of romiplostim (i.e., exceeding the approved dose); three patients had a suspected ADR of renal disorder (non-serious), neutrophil count increased (non-serious), and platelet count decreased (serious).

**Table 2:** Details of romiplostim doses administered to patients in the safety analysis set

Parameter	Safety analysis set (*n* = 1622)
Duration of administration	*n* = 1622
Median (range), days	190 (1, 1203)
Number of administrations	*n* = 1606
Median (range)	24 (1, 110)
Maximum dose	*n* = 1600
Mean ± SD, μg/kg	4.84 ± 3.06
Median (range), μg/kg	4.00 (0.09, 17.50*)
Most frequent dose	*n* = 1600
Mean ± SD, μg/kg	4.03 ± 2.91
Median (range), μg/kg	3.00 (0.09, 13.00)

*SD* standard deviation

*25 patients had received at least one maximum dose of > 10 μg/kg of romiplostim; three patients had a suspected adverse drug reaction of renal disorder (non-serious), neutrophil count increased (non-serious), and platelet count decreased (serious)

### Safety endpoints

The reasons for discontinuation of romiplostim and the major AEs/suspected ADRs that resulted in the discontinuation, dosage at discontinuation, and platelet counts at discontinuation are summarised in Table 3. A total of 64.00% (1038/1622) of patients discontinued romiplostim within 2 years of treatment, and around half of those patients (579/1038 patients) discontinued within 12 weeks. The mean ± standard deviation (SD) dose of romiplostim at the time of discontinuation was 3.87 ± 2.94 μg/kg, and the mean ± SD platelet count was 9.44 ± 13.12 × 10^4^/µL. In this survey, treatment discontinuation with romiplostim occurred in 14.92% of patients due to AEs, and in 6.47% of patients due to suspected ADRs. The most common AEs leading to discontinuation were pneumonia (2.59%), white blood cell (WBC) count increased (1.36%), sepsis (1.17%), anaemia (0.99%), and cerebral haemorrhage (0.99%). The most common suspected ADRs that led to the discontinuation of romiplostim included WBC count increased (0.43%) and cerebral haemorrhage (0.37%). In this survey, 12.45% of patients discontinued treatment with romiplostim because of “inadequate effectiveness”, with a mean ± SD dose of romiplostim at discontinuation being 6.09 ± 3.22 μg/kg and mean ± SD platelet count of 2.63 ± 4.94 × 10^4^/µL; 14.00% of patients discontinued because of “symptom improvement”, with a mean ± SD romiplostim dose and platelet count at the time of discontinuation of 2.33 ± 2.18 μg/kg and 16.38 ± 14.84 × 10^4^/µL, respectively.

**Table 3:** Reasons for discontinuation of romiplostim in the safety analysis set

	Safety analysis set (*n* = 1622)			
	Patients who discontinued within 2 years	Dose at discontinuation within 2 years, μg/kg	Platelet count at discontinuation within 2 years, × 10^4^/µL	Patients who discontinued within 12 weeks
	*n* (%)	(Mean ± SD)	(Mean ± SD)	*n* (%)
Total number of patients who discontinued treatment	1038* (64.00)	3.87 ± 2.94	9.44 ± 13.12	579* (35.70)
AEs leading to discontinuation^#^	242^†^ (14.92)	4.12 ± 2.83	6.73 ± 13.73	138^#^ (8.51)
Pneumonia	42 (2.59)	NA	NA	NA
White blood cell count increased	22 (1.36)	NA	NA	NA
Sepsis	19 (1.17)	NA	NA	NA
Anaemia	16 (0.99)	NA	NA	NA
Cerebral haemorrhage	16 (0.99)	NA	NA	NA
Platelet count decreased	13 (0.80)	NA	NA	NA
Neutrophil count increased	11 (0.68)	NA	NA	NA
White blood cell count decreased	11 (0.68)	NA	NA	NA
Symptom improvement	227 (14.00)	2.33 ± 2.18	16.38 ± 14.84	143 (8.82)
Inadequate effectiveness	202 (12.45)	6.09 ± 3.22	2.63 ± 4.94	102 (6.29)
Other	165 (10.17)	3.29 ± 2.53	9.46 ± 8.62	101 (6.23)
Patient’s wish	151 (9.31)	3.74 ± 2.66	12.60 ± 16.12	65 (4.01)
Transfer to another hospital	133 (8.20)	4.27 ± 2.85	12.27 ± 16.85	67 (4.13)
Suspected ADRs leading to discontinuation	105 (6.47)	NA	NA	NA
White blood cell count increased	7 (0.43)	NA	NA	NA
Cerebral haemorrhage	6 (0.37)	NA	NA	NA
Lost to follow-up	35 (2.16)	3.11 ± 2.43	8.14 ± 7.40	17 (1.05)

*ADR* Adverse drug reaction; *AE* adverse event; *NA* not analysed; *SD* standard deviation

*Duplication was not allowed

^#^ > 10 patients

^†^Including duplicates

The priority survey items of worsening of thrombocytopenia and haemorrhage-related events after discontinuation of romiplostim were evaluated. Of the 1038 patients who discontinued treatment with romiplostim within 2 years, 4.43% (46/1038 patients) had thrombocytopenia recurrence after discontinuation, 80.44% (835/1038 patients) had no recurrence, and 15.13% (157/1038 patients) were unknown. Haemorrhage-related events occurred after discontinuation of romiplostim in 6.07% (63/1038 patients), did not occur in 80.83% (839/1038 patients), and 13.10% (136/1038 patients) of the cases were unknown.

Table 4 highlights the occurrence of AEs and suspected ADRs in the safety analysis set. This survey reported suspected ADRs resulting in death in 2.22% (36/1622) of patients (40 events). Of these, the MedDRA system organ class (SOC) of ‘Infections and infestations’ included pneumonia (*n* = 2) and sepsis, influenza, and *Pneumocystis jirovecii pneumonia* (*n* = 1, each).

**Table 4:** Adverse events and suspected adverse drug reactions in the safety analysis set

Safety parameter	Safety analysis set (*n* = 1622) *n* (%)
AEs	964 (59.43)
SAEs	546 (33.66)
AEs leading to death	237 (14.61)
SAEs (other than death)	309 (19.05)
AEs leading to discontinuation	242 (14.92)
Suspected ADRs (> 1.0%)	438 (27.00)
White blood cell count increased	51 (3.14)
Headache	30 (1.85)
Platelet count decreased	29 (1.79)
Anaemia	27 (1.66)
Malaise	21 (1.29)
Neutrophil count increased	19 (1.17)
Suspected serious ADRs	153 (9.43)
Suspected ADRs resulting in death	36 (2.22)
Cerebral haemorrhage	6 (0.37)
Death*	6 (0.37)
Platelet count decreased	3 (0.18)
Gastrointestinal haemorrhage	2 (0.12)
Myelodysplastic syndrome	2 (0.12)
Cardiac failure	2 (0.12)
Pneumonia	2 (0.12)
Suspected serious ADRs not resulting in death	117 (7.21)
Suspected ADRs leading to discontinuation	105 (6.47)

*ADR* Adverse drug reaction; *AE* adverse event; *SAE* serious adverse event

*Data stating the cause of death in all 6 cases was unknown

Haemorrhagic suspected ADRs were also evaluated as priority survey items. Haemorrhagic suspected ADRs of romiplostim included non-serious and serious haemorrhage. Non-serious/serious haemorrhage occurred in 3.27% (53/1622) of patients. Mean ± SD platelet count at the onset of non-serious/serious haemorrhage was 2.77 ± 4.58 × 10^4^/µL, and 85.07% of these patients had a platelet count of < 5.0 × 10^4^/µL. In these patients, the median onset date was 70.5 (range, 1–680) days. Serious haemorrhage occurred in 1.97% (32/1622) of patients, and 82.93% of patients with serious haemorrhage had a platelet count of < 3.0 × 10^4^/µL. In the safety analysis set (*n* = 1622), the mean ± SD dose of romiplostim at which haemorrhagic suspected ADRs were observed was 4.31 ± 2.88 μg/kg, which was similar to the most frequent romiplostim dose (4.03 ± 2.91 μg/kg) (Table 2).

Thromboembolic suspected ADRs of romiplostim were another priority survey item evaluated. Fifty-eight events occurred in 3.08% (50/1622) of patients. Of these, platelet counts were available for 50 events; 30.00% (15/50) of which occurred at platelet counts of ≥ 40.0 × 10^4^/µL, 16.00% (8/50) at 20.0–40.0 × 10^4^/µL, 46.00% (23/50) at 5.0–20.0 × 10^4^/µL, and 8.00% (4/50) at < 5.0 × 10^4^/µL. The highest incidence of thromboembolism was observed in 46.00% (23/50) of events with platelet counts between 5.0 and 20.0 × 10^4^/µL, followed by 30.00% (15/50) of events with platelet counts over 40.0 × 10^4^/µL (Table 5). For these thromboembolic events, the median onset date was 118 (range, 9–707) days, 36.21% (21/58) of events occurred within 84 days (12 weeks) from the start of romiplostim administration, and 82.76% (48/58) of events occurred within 364 days (52 weeks). In the safety analysis set (*n* = 1622), the mean ± SD dose of romiplostim at which thromboembolic suspected ADRs were observed was 4.12 ± 2.75 μg/kg, which was similar to the most frequent mean dose (4.03 ± 2.91 μg/kg) (Table 2).

**Table 5:** Thromboembolic events in the safety analysis set

Thromboembolic events	*n* (%)
Number of events with available platelet counts	50 (100.00)
≥ 40.0 × 10^4^/µL	15 (30.00)
20.0–40.0 × 10^4^/µL	8 (16.00)
5.0–20.0 × 10^4^/µL	23 (46.00)
< 5.0 × 10^4^/µL	4 (8.00)

Increased bone marrow reticulin or myelofibrosis occurring in the safety analysis set was also evaluated. Increased bone marrow reticulin or myelofibrosis AEs and suspected ADRs were reported in 0.49% (8/1622) and 0.37% (6/1622) of patients, respectively; suspected ADRs included reticulin increased (*n* = 3), myelofibrosis (*n* = 2), and bone marrow reticulin fibrosis (*n* = 1) (Table 6). For events of increased bone marrow reticulin or myelofibrosis, the median onset date was 237 (range, 5–547) days, 42.86% (3/7) of events occurred within 84 days (12 weeks) from the start of romiplostim administration, and 85.71% (6/7) of events occurred within 364 days (52 weeks). No cases of irreversible myelofibrosis were identified from the assessment of these six individual case survey forms.

**Table 6:** Adverse events of special interest in the safety analysis set

Adverse event	Safety analysis set (*n* = 1622)	
	Adverse events *n* (%)	Suspected ADRs *n* (%)
Increased bone marrow reticulin or myelofibrosis	8 (0.49)	6 (0.37)
Reticulin increased	NA	3 (0.18)
Myelofibrosis	NA	2 (0.12)
Bone marrow reticulin fibrosis	NA	1 (0.06)
MDS-related haematological malignancies	15 (0.92)	8 (0.49)
MDS	NA	5 (0.31)
MDS transformation	NA	1 (0.06)
Blast cell counts increased	NA	1 (0.06)
Blast cell proliferation	NA	1 (0.06)
Non-MDS-related haematological malignancies	24 (1.48)	4 (0.25)
Myelofibrosis	NA	2 (0.12)
Plasma cell myeloma	NA	1 (0.06)
Acute leukaemia	NA	1 (0.06)

*ADR* Adverse drug reaction; *NA* not applicable; *MDS* myelodysplastic syndrome

Haematological malignancies and MDS occurring in the safety analysis set were also evaluated. MDS-related AEs and suspected ADRs were reported in 0.92% (15/1622) and 0.49% (8/1622) of patients, respectively. Suspected ADRs included MDS (*n* = 5) and MDS transformation, blast cell counts increased, and blast cell proliferation (*n* = 1, each) (Table 6). For MDS-related haematological malignancies, the median onset date was 128 (range 1–662) days, 42.85% (6/14) of events occurred within 84 days (12 weeks) from the start of romiplostim administration, and 85.71% (12/14) of events occurred within 364 days (52 weeks). AEs and suspected ADRs of non-MDS-related haematological malignancies were reported in 1.48% (24/1622) and 0.25% (4/1622) of patients, respectively. Suspected ADRs included myelofibrosis (*n* = 2), and plasma cell myeloma and acute leukaemia (*n* = 1, each) (Table 6). For non-MDS-related haematological malignancies, the median onset date was 204 (range 15–573) days, 36.00% (9/25) of events occurred within 84 days (12 weeks) from the start of romiplostim administration, and 76.00% (19/25) of events occurred within 364 days (52 weeks). In this survey, we took into account confounding factors and the potential influence of other factors (age, comorbidities, other treatments, complications). After analysing the reported cases, none showed a reasonable possibility of a causal relationship to romiplostim.

### Effectiveness endpoints

**Fig. 2:**
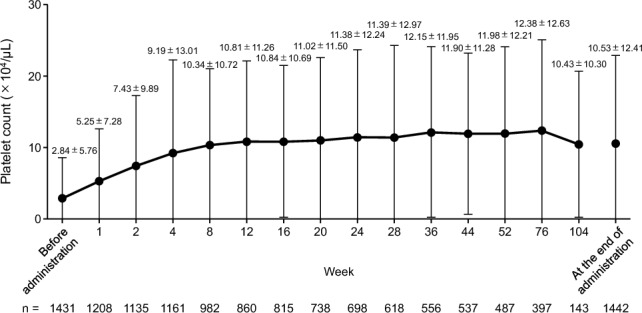
Mean changes in platelet counts from the start of romiplostim administration to 104 weeks (2 years), excluding platelet counts affecting the effectiveness of romiplostim. Data are mean ± standard deviation

Figure 2 shows mean changes in platelet counts from the start of romiplostim administration to 104 weeks (2 years) excluding platelet counts affecting effectiveness. The mean ± SD platelet count before initiation of romiplostim treatment was 2.84 ± 5.76 × 10^4^/µL, which gradually increased to 9.19 ± 13.01 × 10^4^/µL for the first 4 weeks, and ranged from 10.34 ± 10.72 to 12.38 ± 12.63 × 10^4^/µL from 8 to 104 weeks of romiplostim treatment. The proportions of patients with platelet response (doubling of the baseline platelet counts before romiplostim administration and platelet counts ≥ 5.0 × 10^4^/µL) were 60.03% at 24 weeks, 60.25% at 52 weeks, 63.89% at 76 weeks, and 64.53% at 104 weeks.

## Discussion

This large observational survey was conducted for 2 years in all patients treated with romiplostim in real-world clinical settings in Japan. The dose of romiplostim remained below 4 μg/kg in nearly 50% of patients, similar to that reported in earlier clinical trials [9].

Romiplostim was discontinued by 14.00% patients because of symptom improvement. At the time of discontinuation, the mean ± SD dose of romiplostim was 2.33 ± 2.18 μg/kg, and the mean ± SD platelet count was 16.38 ± 14.84 × 10^4^/µL. The results of this survey support the recommendations by the Japanese guidelines for treating chronic ITP in adult patients [18], suggesting that, considering the platelet count variations in this group of patients, a reduced TPO-RA dose may be considered if platelet counts remain > 10.0 × 10^4^/µL.

Based on the results of the approval review, it was considered necessary to carefully monitor platelet counts, especially after discontinuation, wherein there is a definite decline in the platelet count; rapid changes in the platelet count may increase the risk of haemorrhage [10]. Of the 1038 patients in this survey who discontinued romiplostim within 2 years, 4.43% (46/1038 patients) had recurrent thrombocytopenia, and 6.07% (63/1038 patients) had haemorrhage after discontinuation of romiplostim. In this survey, we did not collect information on the treatment details after the discontinuation of romiplostim; hence, no further analyses were performed. The Japanese package insert of romiplostim provides the following cautionary statement: “discontinuation of romiplostim may cause thrombocytopenia when romiplostim is discontinued; the complete blood count (erythrocytes, leukocytes, and platelets) should be monitored frequently for approximately 4 weeks after discontinuation” [4]. In this survey, because most patients did not experience recurrent thrombocytopenia or haemorrhage after discontinuation of romiplostim, we considered that appropriate alternative treatments with existing drugs were administered after romiplostim therapy.

The common AEs that led to the discontinuation of romiplostim were pneumonia, WBC count increased, and sepsis, in this survey. Most of the discontinuations due to these AEs were reported as not related to romiplostim in forms completed by investigators at the survey sites. In addition, suspected ADRs related to ‘Infections and infestations’ that resulted in death were observed in five patients and included pneumonia and sepsis, influenza, and *P. jirovecii pneumonia*. The causal relationship of these events and romiplostim could not be ruled out, including those of unknown causation. However, there were some confounding factors, such as the following: in this survey, 56.97% of patients were aged ≥ 65 years at the start of romiplostim administration; 84.40% used corticosteroids, and 17.76% had undergone splenectomy. Patients with ITP have confounding factors such as disease duration, comorbidities, and surgical history. Because most treatments of ITP use immunosuppression, patients are more susceptible to serious infections or death [19–22]. Because long-term corticosteroid administration compromises immunity towards infections, particular attention should be paid to opportunistic infections such as that with *P. pneumonia* [23].

The Japanese guidelines for treating chronic ITP in adults advise maintaining a platelet count of ≥ 3.0 × 10^4^/µL to prevent serious haemorrhage [18]. In this survey, 82.93% of events of serious haemorrhage were associated with a platelet count < 3.0 × 10^4^/µL. The 85.07% of events with not only serious but also non-serious haemorrhage were associated with a platelet count < 5.0 × 10^4^/µL. The findings of the phase 3 ITP long-term clinical trial safety set, which includes data from phase 3 clinical trials and long-term extension studies, showed a higher incidence of nasal, petechial, gingival, and macular bleeding with platelet count < 5.0 × 10^4^/µL [10]. These results suggest the need for maintaining platelet counts at ≥ 5.0 × 10^4^/µL to control serious and non-serious haemorrhage.

The occurrence of thromboembolic suspected ADRs of romiplostim by platelet count at the time of onset were 8.00% at < 5.0 × 10^4^/µL, 46.00% at 5.0–20.0 × 10^4^/µL, 16.00% at 20.0–40.0 × 10^4^/µL, and 30.00% at ≥ 40.0 × 10^4^/µL. In 718 patients with chronic ITP from the safety analysis sets of clinical trials, the mean ± SD platelet count at the time of onset of adverse events related to thrombosis/thromboembolism was 17.22 ± 21.49 × 10^4^/µL in the romiplostim-treated patients and 15.08 ± 17.06 × 10^4^/µL in the non-romiplostim-treated patients. Even though the platelet count and the time of onset of the AEs were mostly considered unrelated, some patients experienced thromboembolic-related AEs when the platelet count increased immediately after, or excessively above, the discontinuation criterion (40.0 × 10^4^/µL) [10]. The safety of romiplostim was evaluated in patients who received romiplostim at least once in the safety sets of the phase 3 ITP long-term clinical trials and Japanese and foreign long-term extension studies (romiplostim-treated, 151; non-romiplostim-treated, 54). Patients with platelet counts between 20.0 × 10^4^/µL and 40.0 × 10^4^/µL did not have an increased incidence of thrombosis/thromboembolism events per 100 weeks [10]. The Japanese package insert for romiplostim recommends reducing the dose by 1 µg/kg once the platelet count exceeds 20.0 × 10^4^/µL and that romiplostim should be withdrawn if the platelet count exceeds 40.0 × 10^4^/µL [4]. Large cohort studies have demonstrated an increase in arterial and venous thrombosis in patients with ITP [24–26]. The risk of thrombosis/thromboembolism increases with an increase in platelet count beyond the normal range. However, because thromboembolism can occur even when platelet counts are within the normal range, it is essential to carefully monitor the development of thrombosis/thromboembolism irrespective of platelet count [4]. In this survey, 25 patients had received the highest dose of > 10 μg/kg, of which two had suspected non-serious ADRs (renal disorder and neutrophil count increased), and one had a suspected serious ADR (platelet count decreased). In Japan, the approved maximum dose of romiplostim is 10 μg/kg [4]. The Japanese package insert of romiplostim highlights a risk of thromboembolism due to an excessive increase in platelet count resulting from romiplostim overdose [4]. Of the suspected ADRs observed in this survey that corresponded to thromboembolism, none of the patients’ single dose of romiplostim exceeded 10 μg/kg at the onset of the thromboembolic suspected ADR.

This survey recorded AEs and suspected ADRs of increased bone marrow reticulin or myelofibrosis; no case of irreversible myelofibrosis was reported. Bone marrow reticulin has been reported in > 70% of healthy adults [14]. It has been reported in nearly two-thirds of patients with ITP who have not received romiplostim [27] and has been further substantiated in multiple clinical trials [9, 10, 13]. The Japanese package insert suggests periodical monitoring of peripheral blood images, complete blood count, and reticulocyte count before and after starting romiplostim [4].

Although this survey recorded AEs and suspected ADRs of haematological malignancies and MDS, no case of reasonable possibility of a causal relationship to romiplostim was reported. The proliferation of myeloid tumour cells via TPO-RAs occurs through the stimulatory action of the TPO receptor on the surface of the bone marrow cells [10]. An international, phase 2, multicentre, randomised, placebo-controlled, double-blind clinical trial in severe thrombocytopenia-related to MDS (International Prognostic Scoring System low or Intermediate-1) was stopped because of a higher risk of acute myeloid leukaemia transformation with romiplostim versus placebo [28]. The occurrence of haematopoietic tumours has also been reported in patients with chronic ITP [9] and are described in the Japanese package insert of romiplostim [4].

Furthermore, the effectiveness outcomes of this survey are also in accordance with previous clinical study [8]. A long-term extension study examined adult Japanese patients with chronic ITP who participated in domestic phase 2 and phase 3 clinical trials of romiplostim. The dosage of romiplostim was adjusted to achieve platelet counts of 5.0–20.0 × 10^4^/µL and remained stable until the end of the study [8]. In this survey, romiplostim was started based on the approved dose in Japan [4], and a stable therapeutic effect was observed from 8 to 104 weeks of treatment.

### Limitations

A major limitation of this survey is the difference between data collected in clinical trials and observational studies. In a Use-Results Survey, AEs are collected on a physician-reported basis because it is conducted in accordance with GPSP and not the Good Clinical Practice ordinance. The frequency of AEs in such a survey is expected to be underestimated because direct access to medical records and source data verification is not required, as it is in clinical trials, and data are not rigorously collected. However, if the sponsor suspects that an AE has not been reported, the sponsor verifies it with the physician via a query. The reported rates of AEs or suspected ADRs in surveillance studies tend to be lower than those in clinical trials, especially those not requiring therapeutic intervention. Moreover, only Japanese patients were included in this survey, limiting the generalizability of the findings to other populations.

## Conclusions

This post-marketing survey demonstrated suspected ADRs similar to those already listed in package inserts and risk management plans. The findings in this survey did not indicate a significant decrease in effectiveness or a significant change in the incidence of suspected ADRs. Thus, this survey assessing patients treated with romiplostim in Japanese real-world settings revealed no specific concerns regarding the safety or effectiveness of romiplostim. The present findings demonstrate that the risk–benefit balance of romiplostim remains favourable in patients with chronic ITP.

## Supplementary Information

Below is the link to the electronic supplementary material.

Supplementary file1 (DOCX 30 KB)

## Data Availability

The anonymized data underlying the results presented in this manuscript may be made available to researchers upon submission of a reasonable request to the corresponding author.
