# Supplementary material for: Long-term safety and effectiveness of romiplostim for chronic idiopathic thrombocytopenic purpura in real-world settings
Source: Int J Hematol. 2024 Sep 29;120(6):665–74. doi: 10.1007/s12185-024-03847-4 (PMC11588964; doi:10.1007/s12185-024-03847-4)
Supplement: Supplementary file 1 — Supplementary file1 (DOCX 30 KB) [file 12185_2024_3847_MOESM1_ESM.docx]

**Supplementary Table**

**Supplementary Table 1** Primary disease in off-label cases (safety analysis set)

|  | **Number of patients** |
| --- | --- |
| Immune thrombocytopenia | 47 |
| Thrombocytopenia | 9 |
| Myelodysplastic syndrome | 8 |
| Aplastic anaemia | 5 |
| Platelet count decreased | 4 |
| Systemic lupus erythematosus | 4 |
| Wiskott-Aldrich syndrome | 3 |
| Evans syndrome | 2 |
| Acute myeloid leukaemia | 2 |
| Hepatitis C | 1 |
| Castleman's disease | 1 |
| Lymphoma | 1 |
| Hepatic cirrhosis | 1 |
| Plasma cell myeloma | 1 |
| Platelet function tests abnormal | 1 |
| Bone marrow failure | 1 |
| Mucinous adenocarcinoma of the appendix | 1 |
| Disseminated intravascular coagulation | 1 |
| Pancytopenia | 1 |
| Pancreatic cancer | 1 |

Note: Patients for whom the ‘Preferred Term’ of the disease could not be identified were not included.
